# The G-Protein-Coupled Receptor SRX-97 Is Required for Concentration-Dependent Sensing of Benzaldehyde in *Caenorhabditis elegans*

**DOI:** 10.1523/ENEURO.0011-20.2020

**Published:** 2021-01-13

**Authors:** Nagesh Y. Kadam, Sukanta Behera, Sandeep Kumar, Anindya Ghosh-Roy, Kavita Babu

**Affiliations:** 1 Department of Biological Sciences, Indian Institute of Science Education and Research (IISER) Mohali, Punjab 140306, India; 2 Centre for Neuroscience, Indian Institute of Science, Bangalore 560012, India; 3 National Brain Research Centre, Manesar, Nainwal Mode, Gurgaon 122051, India

**Keywords:** ASH neuron, benzaldehyde, *C. elegans*, SRX-97

## Abstract

The G-protein (heterotrimeric guanine nucleotide-binding protein)-coupled receptors (GPCRs) in the olfactory system function to sense the surrounding environment and respond to various odorants. The genes coding for olfactory receptors in *Caenorhabditis elegans* are larger in number in comparison to those in mammals, suggesting complexity in the receptor-odorant relationships. Recent studies have shown that the same odorant in different concentrations could act on multiple receptors in different neurons to induce attractive or repulsive responses. The ASH neurons are known to be responsible for responding to high concentrations of volatile odorants. Here, we characterize a new GPCR, SRX-97. We found that the *srx-97* promoter drives expression specifically in the head ASH and tail PHB chemosensory neurons of *C. elegans*. Moreover, the SRX-97 protein localizes to the ciliary ends of the ASH neurons. Analysis of clustered regularly interspaced short palindromic repeats (CRISPR)-based deletion mutants of the *srx-97* locus suggests that this gene is involved in recognition of high concentrations of benzaldehyde. This was further confirmed through rescue and neuronal ablation experiments. Our work brings novel insights into concentration-dependent receptor function in the olfactory system, and provides details of an additional molecule that helps the animal navigate its surroundings.

## Significance Statement

Although G-protein-coupled receptors (GPCRs) have been known to function as chemosensory receptors, the expression pattern and function of a large number of GPCRs remains unknown. This work sheds light on the expression pattern of an uncharacterized GPCR, SRX-97. Our work shows that this protein is expressed very specifically in two sensory neuron pairs in the head and tail region and is required for concentration dependent sensing of odors in *Caenorhabditis elegans*.

## Introduction

Animals sense a wide range of volatile and water-soluble chemicals through their olfactory system. The olfactory system consists of several neurons that express different sets of seven-transmembrane G-protein-coupled receptors (GPCRs). The odorant binds to the GPCRs, activating distinct intracellular signaling pathways and thus directing the animal’s response to different external cues (for review, see Katritch et al., 2013; Erlandson et al., 2018).

*Caenorhabditis elegans* are soil-dwelling animals that possess well-developed chemosensory systems for their survival. They perceive their environment through various sensory neurons to find food sources, mates, and to escape from dangerous conditions. In *C. elegans,* 13 pairs of chemosensory neurons carry out the majority of chemosensation as they express around 1300 functional chemosensory GPCRs (csGPCRs; Robertson and Thomas, 2006; Vidal et al., 2018). This diversity of csGPCRs allows the animal to discriminate between different odors. Thus, the specific expression of any GPCR or combined expression of different GPCRs on a specific neuron or in multiple neurons can modulate the animal’s perception toward the same odorant.

The olfactory neurons that are involved in sensing a large number of attractive cues are the AWA and AWC neurons. These two pairs of neurons are involved in chemotaxis to various chemicals like diacetyl (DA), isoamyl alcohol (IAA), pyrazine, benzaldehyde, and butanone (Bargmann et al., 1993; Troemel et al., 1995; Colbert et al., 1997). The avoidance behavior toward the repellents nonanone and 1-octanol is mediated through the sensory neurons AWB, ASH, and ADL (Troemel et al., 1997; Chao et al., 2004). Besides this, many volatile chemicals detected by olfactory neurons could act as attractants at low concentrations and repellents at high concentrations (Yoshida et al., 2012). For example, at low concentrations, DA is sensed by the GPCR ODR-10, in the AWA neuron acting as an attractant (Sengupta et al., 1996), whereas at high concentration it is sensed by the SRI-14 GPCR in the ASH neurons and acts as a repellent (Taniguchi et al., 2014). Additionally, ASH neurons are polymodal neurons involved in avoidance behaviors toward different nociceptive signals, like noxious chemicals, nose touch, hyperosmolarity, and volatile repellents (Hilliard et al., 2005; Bargmann, 2006). The ASH neurons convey information through multiple receptors. For example, nose touch has been shown to be detected by transient receptor potential (TRP) channel proteins like OSM-9 and OCR-2 (Colbert et al., 1997; Tobin et al., 2002), while hyperosmolarity is detected by OSM-10 (Hart et al., 1999). The ASH neurons forms strong synaptic connections with the AVA command interneurons, which regulates the backward locomotion of *C. elegans* (Gray et al., 2005; Zheng et al., 2012; Pokala et al., 2014; Bhardwaj et al., 2018, 2020). Thus, activation of ASH neurons can affect backward locomotion or avoidance behaviors in *C. elegans*.

ASH neurons are also reported to be involved in sensing undiluted or high concentrations of benzaldehyde (Troemel et al., 1995; Walker et al., 2009; Aoki et al., 2011; Taniguchi et al., 2014). Here, we show that SRX-97 is expressed in the ASH neurons. We have used the clustered regularly interspaced short palindromic repeats (CRISPR)/Cas9 method for genome editing and have made a deletion in the *srx-97* gene locus, generating a null mutation in *srx-97*. The *srx-97* mutants present defects in chemotaxis behavior, more specifically toward high concentrations of benzaldehyde. Moreover, the mutant phenotype could be rescued by both endogenous and neuron-specific expression of the wild-type (WT) *srx-97* gene, suggesting concentration-dependent behavioral plasticity for odors in *C. elegans* through the SRX-97 GPCR.

## Materials and Methods

### *C. elegans* strains and maintenance

All *C. elegans* strains were maintained on nematode agar growth media (NGM) plates seeded with OP50 *Escherichia coli* at 20°C under standard conditions (Brenner, 1974). The *C. elegans*, N2 (Bristol strain) was used as the WT control, and the mutant strains CX2205 *odr-3* (*n2150*) V, CX10 *osm-9* (*ky10*) IV, NL792 *gpc-1*(*pk298*) X, RB2464 *tax-2* (*ok3403*) I and VC3113 *tax-4* (*ok3771*) III, as well as the AWC ablated strain PY7502 *oyIs85* (P*ceh-36*::TU#813 + P*ceh-36*::TU#814 + P*srtx-1*::GFP + P*unc-122*::DsRed, TU#813 and TU#814 are split caspase vectors) used in this study were obtained from the *Caenorhabditis* Genetic Centre (CGC). Double mutants were made through standard genetic procedures and verified using PCR. The list of primers used for PCR verification in this study is tabulated in Table 1. The strains used in this study are listed in Table 2.

**Table 1:** Primers used in this study

Primer number	FP/RP	Primer sequence	Gene name	Vector backbone
NK181 NK196	FP RP	atcagcatgcatcttgaaaacctcaatcgaaccag ctctctcccgggggacatatcttgaaagtttggaatggag	P*srx-97*	*pPD49.26*_mCherry
NK214 NK196	FP RP	ctctctcgcatgcggtaagttttgcagtctaggcag ctctctcccgggggacatatcttgaaagtttggaatggag	P*srx-97*	*pPD49.26_mCherry*
NK259 NK260	FP RP	ctctctgcatgcggaaccgtatttttgtgcaatagtcg ctctctcccggggcaagatgaaatttccaaaaaagtttattgatatgg	P*osm-10*	*pPD95.75*
NK262 NK263	FP RP	ctctctgcatgcgccaaaactgctgaacttttg ctctctcccgggcttctgtagaaatttcaagactgatcac	P*srb-6*	*pPD95.75*
NK197 NK279	FP RP	ctctctcccgggatgtccttatcgaattggacgc ctctctggtaccttcaacatgatcctattcaagtttggtatttttc	*srx-97_UTR*	*pPD49.26*
NK197 NK254	FP RP	ctctctcccgggatgtccttatcgaattggacgc ctctctggtacctcaaaatgtgactgttaaaactgtgactt	*srx-97* gene	*pPD49.26*_mCherry
gRNA_1		atcaggtctcctcttccccacttatgactattacagttttagagctagaaatagcaag	*srx-97* gene	*pRB1017*
gRNA_2		atcaggtctcctcttaaaattataaggcgtaggcagttttagagctagaaatagcaag	*srx-97* gene	*pRB1017*
Homology amr_1	FP RP	atcatctagatcttccggacgtgaatcttctatc atcagcatgcatcttgaaaacctcaatcgaaccag	*srx-97* gene	*pPD95.75*
Homology amr_2	FP RP	atcagggccccattgcacaactgataagatagtgc atcacttaagttcaacatgatcctattcaagtttggt	*srx-97* gene	*pPD95.75*
NK263 NK264 NK265	EFP IFP RP	ctacagtttagtgcttgccacag tgtcgaaattaaagggtttcgagg gctgtccaacgcaattttcg	*gpc-1*	genotyping
NK303 NK304 NK279	EFP IFP RP	gctaggtggagggctgattg gctaggtggagggctgatta atgggcggtggaagttcg	*osm-9*	genotyping
NK207 NK209	FP RP	ggatagaagattcacgtccggaag tccatgtggggtttgctctg	*srx-97*	genotyping
NK210 NK179	FP RP	cttccaacatgaaaagcactatcttatcag ttcaacatgatcctattcaagtttggt	*srx-97*	genotyping
PRS394 PRS395 PRS396	FP ERP IRP	gcggttcggatacgaaaatacttg gacggagaagtgtatccgttatatc ccatgcgtccgtccctaatcc	*tax-4*	genotyping
PRS442 PRS443 PRS444	FP ERP IRP	cactggcgacgattgtcagatc gttatttccagtagatgtggccacg gtagccaaaatgagttgatctg	*tax-2*	genotyping
AB37 AB38 AB39	FP-WT FP-*glr-1* RP	acctttcggctccgacttg acctttcggctccgactta attgaaatgaccataccacc	*glr-1*	genotyping

FP, forward primer; RP, reverse primer; FP-WT, external forward primer for WT *glr-1* sequence; FP-*glr-1*, external forward primer for *glr-1* sequence in the *glr-1* point mutation line.

**Table 2:** List of strain used in this study

Strain name	Genotype	Source
BAB430 (CGC strain CX2205)	*odr-3 (n2150)*	From CGC (2× outcrossed)
BAB431 (CGC strain CX10)	*osm-9 (ky10)*	From CGC (3× outcrossed)
BAB432 (CGC strain RB2464)	*tax-2 (ok3403)*	From CGC (3× outcrossed)
BAB433 (CGC strain VC3113)	*tax-4 (ok3771)*	From CGC (3× outcrossed)
BAB434 (CGC strain NL792)	*gpc-1 (pk298)*	From CGC (3× outcrossed)
BAB503 (CGC strain KP4)	*glr-1 (n2461)*	From CGC (3× outcrossed)
BAB404	*srx-97*	This study (3× outcrossed)
PY7502	*oyIs85*	From CGC
BAB466	*Psrx-97 (600 bp)::*mCherry (*indEx459*)	This study
BAB467	*Psrx-97::mCherry (indEx460)*	This study
BAB482	*srx-97;* P*srx-97*::SRX-97_ UTR (*indEx462*)	This study
BAB483	*srx-97;* P*osm-10*::SRX-97_UTR (*indEx466*)	This study
BAB462	P*srx-97*::SRX-97::mCherry (*indEx461*)	This study
BAB494	*srx-97*; P*srx-97*::SRX-97::mCherry (*indEx461*)	This study
BAB478	P*srx-97*::SRX-97_ UTR (*indEx462*)	This study
BAB437	P*osm-10*::SRX-97_UTR (*indEx466*)	This study
BAB492	P*srx-97*::mCherry (*indEx460*)*;* P*srb-6::*GFP (*indEx465*)	This study
BAB493	P*srx-97*::mCherry (*indEx460*)*;* P*osm-10::*GFP (*indEx464*)	This study
BAB473	*srx-97; osm-9*	This study
BAB487	*srx-97; odr-3*	This study
BAB488	*srx-97; tax-2*	This study
BAB489	*srx-97; tax-4*	This study
BAB490	*srx-97; gpc-1*	This study
BAB491	*srx-97; oyIs85*	This study
BAB492	*odr-3; oyIs85*	This study

### Rescue constructs and transgenes

All constructs for the rescue of the *srx-97* phenotype were generated using standard cloning methods (Sambrook and Russell, 2001). The *pPD49.26* and *pPD95.75* vectors were used to clone the constructs. The primers used for cloning are indicated in Table 1. The transgenic strains were generated using standard microinjection techniques as described previously (Mello et al., 1991; Mello and Fire, 1995). The *pCFJ90* and *pPD95.75* plasmids were used to amplify or clone mCherry and GFP, respectively. The rescue constructs or promoter fusion constructs were injected at 20–30 ng/μl. P*myo-2*::mCherry (2 ng/μl) or P*unc-122*::GFP (25 ng/μl) were used as co-injection markers. The constructs used in this study are described in Table 3.

**Table 3:** Plasmids used in this study

S. no.	Plasmid ID	Plasmid
1	pBAB459	P*srx-97 (600 bp)*::mCherry
2	pBAB460	P*srx-97*::mCherry
3	pBAB461	P*srx-97*::SRX-97::mCherry
4	pBAB462	P*srx-97*::SRX-97_UTR
5	pBAB464	P*osm-10::*GFP
6	pBAB465	P*srb-6::*GFP
7	pBAB466	P*osm-10*::SRX-97_UTR
8	pBAB472	*srx-97*_homology arms
9	pBAB470	gRNA_1
10	pBAB471	gRNA_2

### Imaging experiments

Young adult animals were used for imaging. The animals were immobilized with 30 mg/ml 2, 3-butanedione monoxamine (BDM) on 2% agarose pads in M9 media. The promoter mCherry images for ASH and PHB were acquired on a Leica SP6 upright laser scanning confocal microscope using the 40× oil-immersion objective lens. Laser lines from He-Ne (594) with HyD detectors were used to image fluorescence in the head and tail regions. All other imaging experiments were performed with oil immersion 40×/1.4, 63×/1.4 or 100×/1.4 plan Apochromat objectives using a Zeiss AxioCam MRm CCD camera on the Zeiss AxioImager Z2 microscope.

### Behavioral assays

#### Chemotaxis assay

The chemotaxis assay was performed using young adult *C. elegans*. Young adult animals were obtained by bleaching gravid adults and incubating the remaining eggs for 72 h (h) at 20°C. All chemotaxis assays were performed with standard 90 mm petriplates containing 15–18 ml of chemotaxis medium (Agar, 1 m MgSO_4_, 1 m CaCl_2_, and 1 m KPO_4_; pH 6.6). Wherever required, odorants were diluted in ethanol and reported as a percent by volume. Modified 90-mm quadrant plate chemotaxis assays were performed as described previously (Bargmann et al., 1993; Margie et al., 2013). Briefly, 5 min before the assay, 1 μl of 0.5 m sodium azide was applied on four spots that were each 3 cm from the loading center. Sodium azide acts as an anesthetic agent to immobilize animals that reach the vicinity of the spot during the assay. A total of 50–150 animals were placed at the center of the plate between the four spots, 2 μl of ethanol were placed at the two-control spots and 2 μl of the test odorant were placed at the two-test spots. After 90 min of chemotaxis, animals within each sector were counted, and the chemotaxis index (C.I.) was calculated as the number of animals in the two test sectors minus the number of animals in the two control sectors, divided by the total number of animals on the plate excluding those that were not moving at the center of the plate (illustrated in Extended Data Fig. 3-1*A*). A positive C.I. indicates an attraction to the chemical, and a negative C.I. indicates a repulsion to the chemical.

#### Assay to evaluate chemotaxis frequency

For analysis of the frequency or number of animals chemotaxing toward the source of benzaldehyde, a modified grid chemotaxis plate was used (Nuttley et al., 2001). The sodium azide was omitted so that animals could leave a spot after an initial approach. This grid consisted of four parallel lines drawn 1 cm apart to divide the plate area into five sectors, with the distance between the second and third lines being 2 cm (illustrated in Fig. 4*A*). Two microliters of benzaldehyde were placed on one small sheet of Parafilm, and the same amount of ethanol was placed on another as a control. The benzaldehyde and ethanol were placed at opposite ends of the plate (6 cm away). After a 60-min time interval, animals were immobilized by cooling the plates for 3 min at −30°C, and the plates were maintained at 4°C until counting. The number of animals in sectors a–d, with the test odorant being in a and d, were counted and the kinetic C.I. was calculated as (number of animals in a + number of animals in b) − (number of animals in c + number of animals in d)/(total number of animals on the plate), yielding a C.I. range between +1.0 and −1.0 (illustrated in Fig. 4*A*). The animals that had crawled up the sides of the plate were excluded from the analysis. The score of 50–150 animals for each plate was used as one data point.

#### Dry drop avoidance assay

A drop of a solution containing the test chemicals [SDS, quinine, CuSO_4_, glycerol, and dihydrocaffeic acid (DHCA)] dissolved in M13 buffer (30 mm Tris, 100 mm NaCl, and 10 mm KCl) was delivered on the agar plate (NGM unseeded) 0.5–1 mm anterior to the moving animals (Hilliard et al., 2002). Once the animal encountered the dry drop of chemical, the head amphid neurons sensed the chemical triggered repulsion/avoidance behavior. The delayed response in seconds from the initial contact to a reversal was calculated in the assay. Videos were recorded for 1 min at 10 frames/s with one to two readings leaving a gap of 20–30 s between each trial. The graphs were plotted by taking the average value from two trials with >30 animals being analyzed for each condition over multiple days. If the animal failed to respond within 6 s, the reversal time was considered as 6 s. Drops of M13 buffer were used as a control where animals as expected did not show robust responses. Glass capillaries (10 mm) pulled by hand on flames to reduce the diameter of the tip were used to deliver the drops. The results were plotted using GraphPad Prism V6 and evaluated using one-way ANOVA. The mean ± SEM were plotted.

#### Nose touch assay

The response to nose touch was analyzed on unseeded plates as described previously (Kaplan and Horvitz, 1993). Briefly, young adult animals were placed on NGM plates and allowed to habituate for 1 min. An eyelash was placed in the path of the forward moving animal, and those who showed a reversal of the body movement on collision with the eyelash were considered as positive responders. The experiment was performed with 20–30 animals per genotype over multiple days. The analysis was performed using 10 trials/animal, and the data are shown as the percentage of positive responders.

#### Aldicarb assay

The aldicarb assays were performed as described previously (Mahoney et al., 2006). Briefly, Aldicarb plates were made the previous day by adding 100 mm stock solution (prepared in ethanol) of aldicarb (Sigma-Aldrich) to molten NGM at a final concentration of 1 mm. Plates were then seeded with OP50 *E*. *coli* and stored in dark at room temperature overnight. For each assay 20–25 young adult animals were transferred on to the aldicarb plates and scored for paralysis every 10 min for up to 120 min. Animals were considered paralyzed when they failed to show body bends following prodding three times on the head.

### Ablation of ASH neurons

ASH neuronal ablation experiments were performed to test the benzaldehyde chemotaxis dependence on this neuron, which was tagged with P*srx-97*::mCherry. L2 staged animals were used for the ablation experiment, as ablations are more effective in early stages (Avery and Horvitz, 1987, 1989; Bargmann and Horvitz, 1991). During ablation and imaging, the animals were immobilized on 5% agarose pads with 0.1-μm-diameter polystyrene beads (00876-15; Polystyrene suspension). The Bruker Corporations ULTIMA setup was used to perform two-photon imaging and ablations simultaneously (Basu et al., 2017). A 60× water immersion objective was used for ablation and imaging experiments, GFP and mCherry were visualized using 920- and 1040-nm lasers. A shot for 60 ms pulsed femtosecond IR laser [pulse width 80 fs, irradiation pulse width: 50 ms, laser point spread function (PSF) 400 nm and *z*-axis PSF-1.5um and wavelength of the laser 720 nm] was used for all ablation experiments. Animals were then examined for successful ablation under a fluorescence microscope. These animals were allowed to grow and recover until they reached the young adult stage. Single animals were then transferred to individual unseeded plates and allowed to habituate for 1 min. Benzaldehyde (concentration of 10^−1^) was filled in the glass capillary having a small opening pore. The filled capillary was held just in front of the anterior region of the forward moving animals. Videos were recorded for 5 min at 10 frames/s with five to six trials leaving a gap of ∼1 min between each reading. Graphs were plotted by taking the average value from five to dix trials, with around 25 animals analyzed for each condition over multiple days. The results were plotted using GraphPad Prism V6 and evaluated using one-way ANOVA. The mean ± SEM was plotted.

### CRISPR/Cas9 mediated deletion of the *srx-97* gene

The CRISPR/Cas9 system was used to create the *srx-97* deletion mutation, as described previously (Dickinson and Goldstein, 2016). The two guide RNAs were designed (Hsu et al., 2013) and cloned separately into the *pRB1017* vector under the *CeU6* promoter. The Cas9 enzyme was expressed from the *pJW1259* vector under the *erf-3* promoter. The selection excision cassette (SEC) containing plasmid *pDD287* was cloned along-with flanking loxP sites into the *pPD95.75* vector as described previously (Dahiya et al., 2019). The resulting plasmid was used to clone homology arms (500–600 bp) using restriction enzyme-based cloning methods.

The plasmid mixture containing repair template (40 ng/μl), sgRNA_1 (10 ng/μl), sgRNA_2 (10 ng/μl), *pJW1259* (50 ng/μl), *pCFJ90* (2.5 ng/μl), and P*vha-6*::mCherry (15 ng/μl) was injected into 20–30 adult hermaphrodite animals (containing four to five eggs) that were kept at 20°C. Hygromycin was added after 60 h of injection, directly on the NGM plate containing *C. elegans*. The hygromycin treated plates were left for 10 d at 20°C. Next, 20–30 non-fluorescent rollers were singled out on regular seeded NGM plates. Once 100% roller progeny were observed on the plates, these plates were kept at 34°C for 3–4 h. Normal moving *C. elegans* were then picked and allowed to produce progeny. The genomic DNA was isolated from these progenies and the desired deletion was confirmed using PCR and sequencing techniques.

### Statistical analysis

All statistical analyses on behavioral assays were performed by using GraphPad Prism version 6.0. The error bars represent SEM. Statistical significance was determined using one-way ANOVA along with the Sidak’s *post hoc* test for multiple comparisons. Asterisks in the graphs indicate that the mean differences were statistically significant. The levels of significance were set as *p <* *0.05, **p <* *0.01, ***p <* *0.001.

## Results

### The P*srx-97*::mCherry transgene presents unique expression in the ASH and PHB chemosensory neurons

csGPCRs are categorized into nine different classes based on their sequence homology with the Rhodopsin class of molecules (Fredriksson et al., 2003; Lagerstrom and Schioth, 2008). The *C. elegans* genome has 1341 genes coding for GPCRs; however, the expression pattern of only 320 genes is known at a single-cell resolution (Robertson and Thomas, 2006; Taniguchi et al., 2014; Vidal et al., 2018). Reports suggest that GPCRs are also expressed in non-neuronal tissues like intestine and are involved in sensing internal cues (Vidal et al., 2018). Some GPCRs change their expression pattern once the animal encounters starvation or dauer-ike conditions (Vidal et al., 2018). Despite a large number of studies on the functional and spatial diversity of GPCRs, the expression pattern and function for a majority of the csGPCRs are still unknown (Robertson and Thomas, 2006; Taniguchi et al., 2014; Vidal et al., 2018).

**Figure 1.**
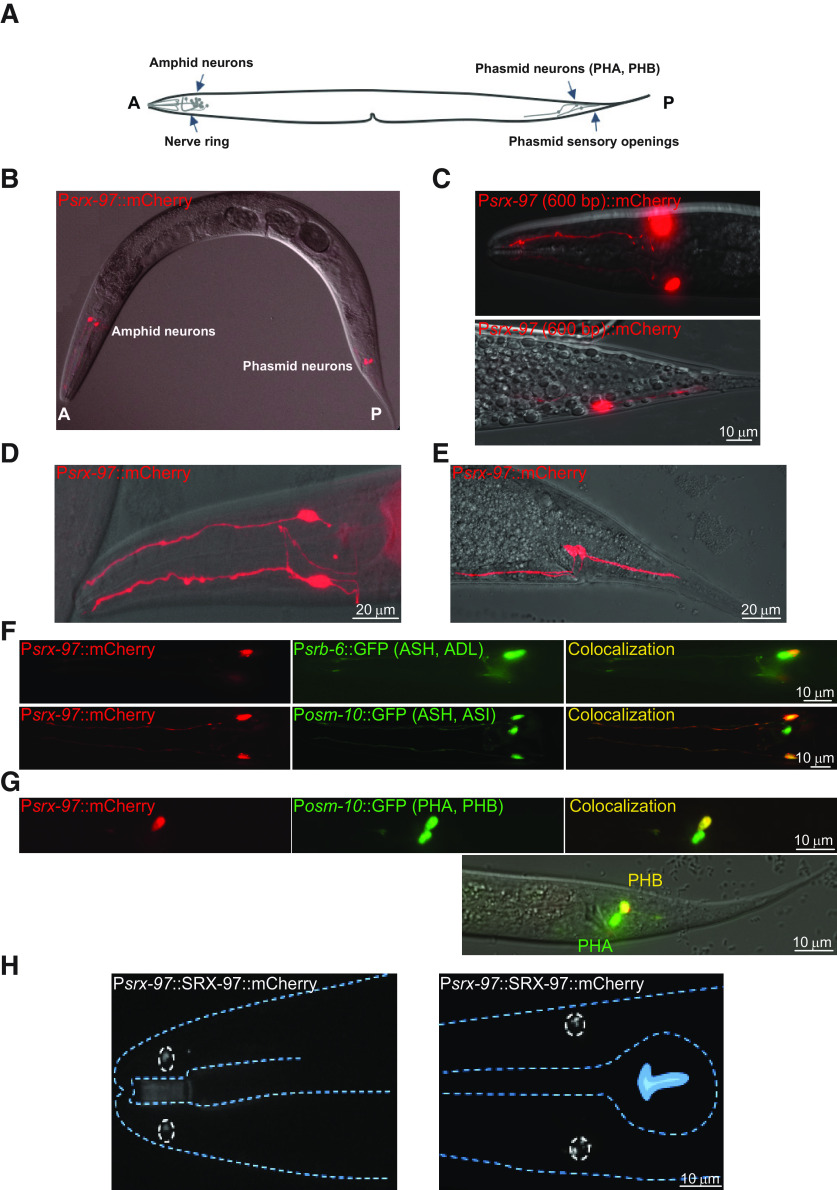
Expression of P*srx-97*::mCherry in ASH and PHB neuron. ***A***, Cartoon image showing the location of the amphid and phasmid neurons in *C. elegans.* ***B***, Expression of the P*srx-97*::mCherry transgenic construct in the whole animal. ***C***, *srx-97* promoter (600 bp) expression in a single pair of amphid and phasmid neurons. ***D***, The expression of the *srx-97* promoter (2 kb) in a pair of amphid neurons and (***E***) phasmid neurons in *C. elegans*. ***F***, Expression of P*srb-6*::GFP and P*osm-10*::GFP in their respective neurons (indicated on the figure) and their co-localization with P*srx-97*::mCherry in the amphid ASH neurons. ***G***, Expression of P*osm-10*::GFP in its respective neurons (indicated on the figure) and their co-localization with P*srx-97*::mCherry in the phasmid PHB neuron. The lower panel indicates a DIC image indicating the position of the PHA and PHB neurons. ***H***, Expression of SRX-97::mCherry in the cell bodies (dotted circles) and SRX-97 localization to the cilium tip of the ASH neurons (between the dotted circles in the figure to the left).

We started this study to gain insight into the function of a previously uncharacterized GPCR, SRX-97. SRX-97 was identified in an aldicarb-based RNAi screen and was found to be hypersensitive to aldicarb (Babu et al., 2011; Babu et al., unpublished data). To determine the expression pattern of SRX-97, a region 2 kb upstream of the predicted translational start codon of the *srx-97* gene along with six base pairs of the first exonic region were used as a promoter to generate the P*srx-97::*mCherry reporter line. In these transgenic animals, mCherry expression was specifically detected in a single pair of head amphid neurons and a single pair of tail neurons (Fig. 1*A*,*B*,*D*,*E*). Moreover, even a 600-bp region upstream of the *srx-97* gene with six base pairs from the first exon showed a similar expression pattern as the 2-kb promoter (Fig. 1*C*). No expression was detected in any other part of the body. Since the amphid and phasmid neurons are involved in chemotaxis, our data suggested that SRX-97 could specifically be involved in these neurons and likely in chemosensory signaling.

Next, we began identifying P*srx-97*:: mCherry expressing neurons based on their cilium morphology, the cell body position in the head and tail region, and colocalization experiments. To uncover the neurons that showed expression of the *srx-97* promoter, we generated the P*srb-6*::GFP transgenic line, which shows expression in the ASH and ADL neurons in the amphid region (Troemel et al., 1995). The P*srx-97::*mCherry line presented colocalization in a single neuron pair with the P*srb-6*::GFP line (Fig. 1*F*, top panel), indicating that P*srx-97*::mCherry could be expressed in either the ASH or the ADL neurons. To conclusively identify the P*srx-97*::mCherry expressing neuron, we generated another transgenic line with P*osm-10*::GFP, which shows expression in the amphid ASH and ASI neurons and the PHA/PHB neurons in the phasmid region (Fig. 1*F*, bottom panel) and (Hart et al., 1999). The colocalization of P*srx-97*::mCherry in a single amphid neuron pair with both marker lines indicated that the *srx-97* promoter drives expression in the ASH neurons. In line with a recent report that suggests that 50% of GPCRs which express in ASH neurons also show expression in the PHB neuron (Vidal et al., 2018), we found that in the tail region, P*srx-97*::mCherry showed colocalization with a pair of phasmid neurons (Fig. 1*G*, top panels indicating colocalization with one phasmid neuron). Based on the orientation of the animal (posterior right and ventral down), this neuron appears to be the PHB neuron (Fig. 1*G*, DIC image in bottom panel).

We then analyzed a SRX-97 translational reporter and found that the P*srx-97*::SRX-97::mCherry transgenic line showed SRX-97 protein localization toward the cilium tip of the ASH neurons (Fig. 1*H*), indicating that this protein may be involved in sensing environmental cues from the surroundings.

### CRISPR/Cas9 mediated deletion of *srx-97*

*C. elegans* have 13 pairs of chemosensory neurons in the anterior amphid and posterior phasmid regions. However, it can detect several different chemical cues ranging from volatile to water-soluble odorants through diverse GPCRs (Robertson and Thomas, 2006; Vidal et al., 2018). Seven percent of the *C. elegans* genome encodes for chemoreceptors. However, only around 900 chemoreceptor genes have been characterized functionally, many through RNAi experiments (Robertson and Thomas, 2006; Taniguchi et al., 2014; Vidal et al., 2018). Hence, only a few mutant lines of GPCRs are available. Our studies have shown that the *srx-97* promoter drives expression in ASH and PHB neurons. This expression pattern raised the possibility that it might function as a receptor for odorant/s. Since no mutant strain was available for this gene, we used the CRISPR/Cas9 based strategy to generate a deletion in the *srx-97* gene.

**Figure 2.**
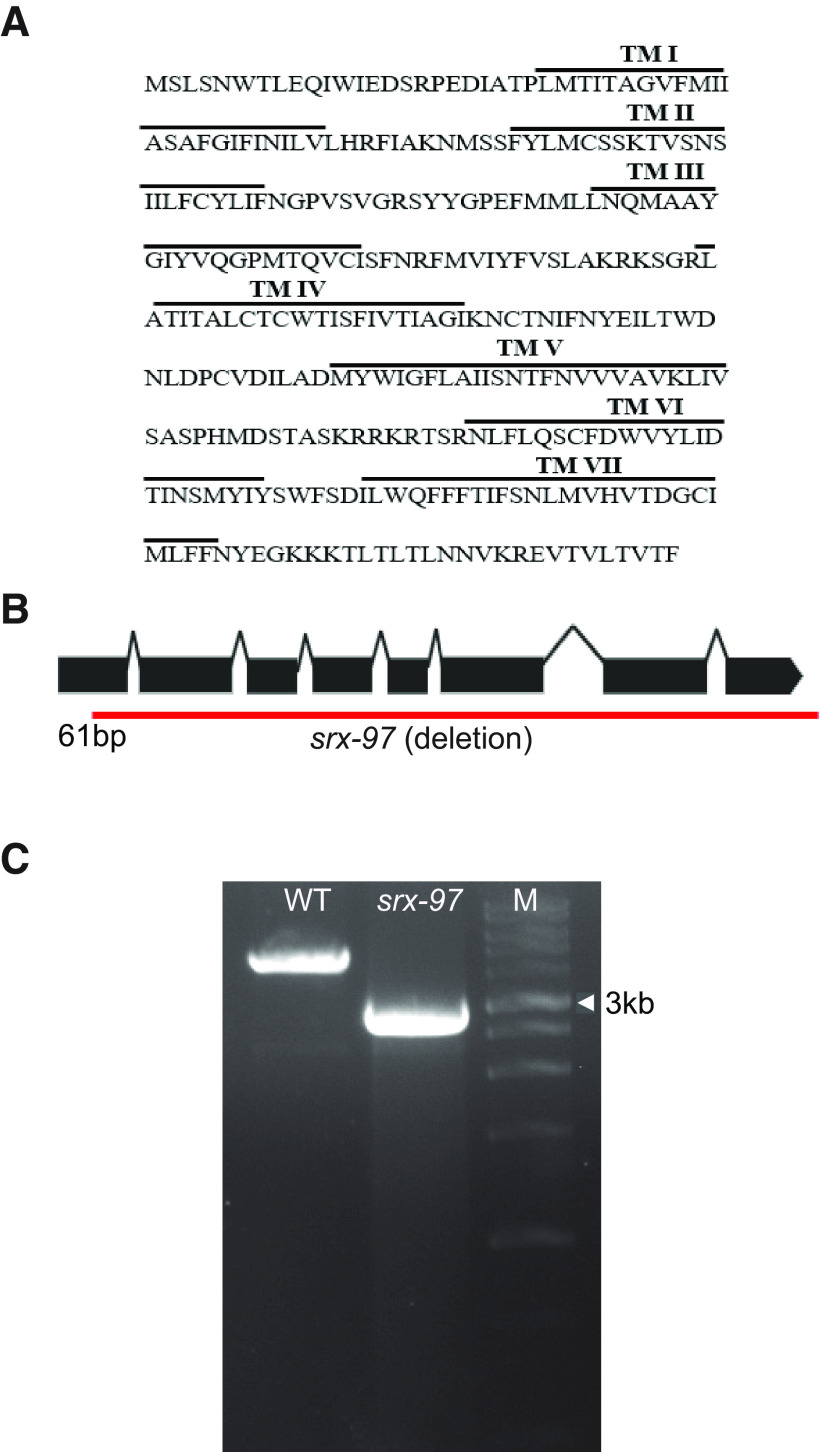
The SRX-97 transmembrane domain and CRISPR/Cas9 generated mutation of *srx-97*. ***A***, Amino acid sequence showing the predicted seven transmembrane domain of SRX-97. ***B***, Exonic structure of the *srx-97* gene with the red line showing the CRISPR/Cas9 deletion obtained. The deletion encompasses the gene from the 61st base pair to the 1895th base pair including part of the 3′ UTR of the gene. ***C***, Amplification of the chromosomal region showing the deletion of the *srx-97* gene (2730 bp) using CRISPR/Cas9 compared with control WT (4566 bp) gene. A 1-kb DNA ladder was used in the line marked Marker (M). Extended Data Figure 2-1 supports this figure.

The SRX-97 GPCR is part of the SRX family of proteins that belong to the SRG superfamily that encodes around 320 genes (Robertson and Thomas, 2006; Vidal et al., 2018). The *srx-97* gene encodes a predicted protein of 317 amino acids (Fig. 2*A*). Hydrophobicity analyses showed that the SRX-97 protein encodes for a seven-transmembrane domain protein, with the characteristic topology of GPCRs (Fig. 2*A*). By using the gene-editing CRISPR/Cas9 technique, we made a complete deletion of the *srx-97* gene (from 61 bp of the first exon to the 3′ UTR region, deleting a 1834-bp sequence; Fig. 2*B*,*C*). We next performed aldicarb assays with the *srx-97* deletion animals and found no significant difference in aldicarb sensitivity between the deletion strain and WT control animals (Extended Data Fig. 2-1*A*). These data, along with the localization and expression pattern of SRX-97, indicated that SRX-97 likely did not function at the neuromuscular junction to cause defects in aldicarb sensitivity. The aldicarb phenotype in the RNAi screen could have been a false positive because of cross-complementation reactions with other GPCRs. Hence, we proceeded to study the role of SRX-97 in other processes.

10.1523/ENEURO.0011-20.2020.f2-1 Extended Data Figure 2-1 SRX-97 is not required for aldicarb-induced paralysis in *C. elegans*. ***A***, Graph of *C. elegans* paralyzing on aldicarb performed with WT and *srx-97* mutant animals. The assay was performed over a course of 2 h, and the percentage of animals paralyzed was plotted every 10 min. There was no significant difference between the percentage of animals paralyzed at any given time point in *srx-97* when compared to the WT control animals. The experiment was performed in triplicate with 20–25 animals assayed per genotype for each experiment. Download Figure 2-1, EPS file.

### Loss of *srx-97* leads to defects in chemotaxis toward benzaldehyde

**Figure 3.**
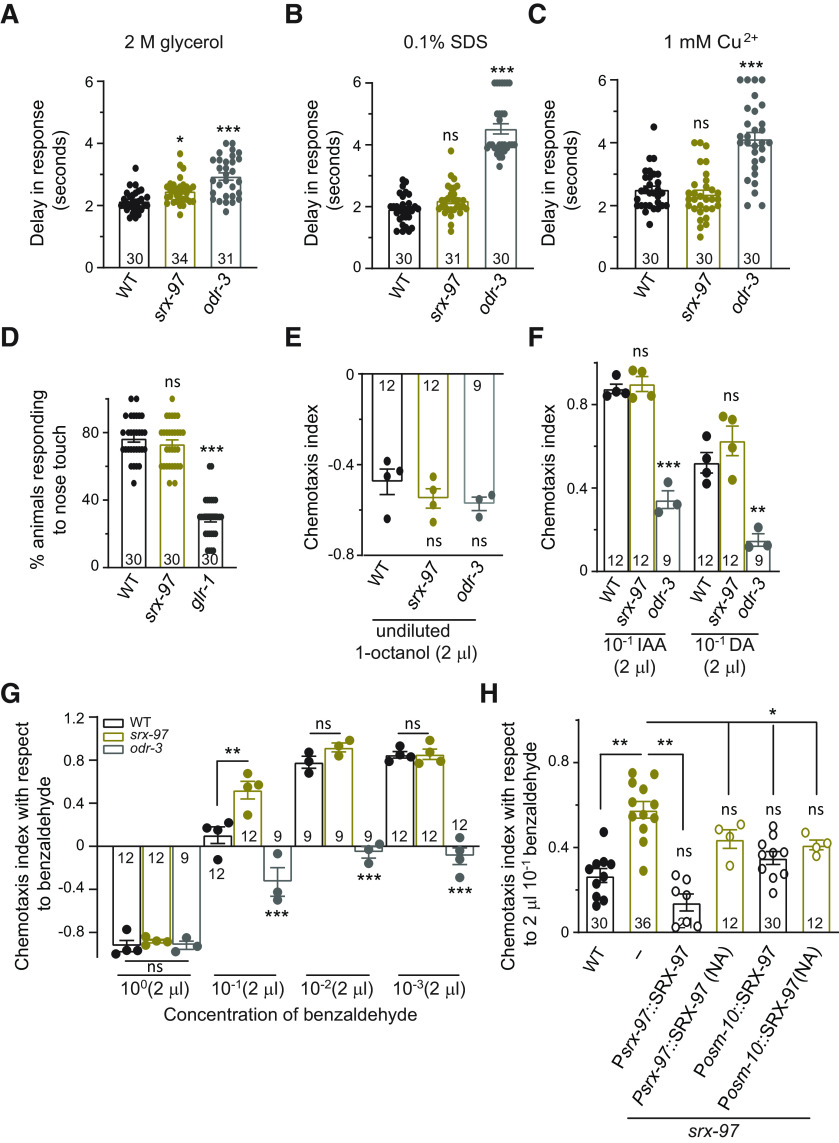
Behavior of *srx-97* mutant animals toward water-soluble and volatile chemicals. ***A***, Graph showing the delay in avoidance response toward a dry spot of 2 m glycerol in WT, *srx-97*, and *odr-3* mutant animals. The number of animals assayed for each genotype is indicated at the base of each plot for panels ***A–D***. ***B***, Graph showing the delay in avoidance toward a dry spot of 0.1% SDS in WT, *srx-97*, and *odr-3* mutant animals. ***C***, Graph showing the delay in avoidance toward a dry spot of 10 mm CuSO_4_ in WT, *srx-97*, and *odr-3* mutant animals. ***D***, Graph showing the percentage of avoidance on nose touch stimuli of WT, *srx-97*, and *glr-1* mutant animals. The numbers at the base of graphs in ***A–D*** indicate the number of animals tested for each genotype. ***E***, Graph indicating the negative chemotaxis indices of WT, *srx-97*, and *odr-3* mutant animals toward the repellent octanol. The assay was done in triplicates over multiple days for all chmotaxis assays. Each dot indicates an assay done in triplicate for all graphs from ***E–H***. ***F***, Chemotaxis indices toward high concentrations (10^−1^) of DA and IAA. ***G***, Chemotaxis indices toward multiple concentrations of benzaldehyde. ***H***, Chemotaxis indices toward high concentrations of benzaldehyde in WT, *srx-97*, and rescue strains of *srx-97*. This rescue experiments used SRX-97 under its own promoter and under the *osm-10* promoter. Animals that did not show expression of the arrays (NA, no array) were used as controls in these experiments. The error bars represent SEM, and statistical significance is represented as “ns” for not significant, *p <* *0.05, **p <* *0.01, ***p <* *0.001. The numbers at the base of each graph from ***E–H*** indicates the total number of times the experiment was performed with 50–150 animals used in each trial. Extended Data Figure 3-1 supports this figure.

ASH is a polymodal neuron that can respond to noxious, mechanical and osmotic stimuli (Kaplan and Horvitz, 1993; Colbert et al., 1997; Hilliard et al., 2004, 2005). To characterize the role of the SRX-97 GPCR in ASH neurons, we examined the response of the *srx-97* mutant line toward several compounds including glycerol, SDS, Cu^2+^, quinine, DHCA, and acetic acid (Kaplan and Horvitz, 1993; Hilliard et al., 2002, 2005). The *srx-97* mutant animals showed minor defects in avoidance behavior toward 2 m glycerol and 100 mm DHCA when compared with the control WT animals (Fig. 3*A*; Table 4). We also tested the behavior of *srx-97* mutants to other chemicals and found that *srx-97* did not show significant differences when compared with WT control animals in their behavior toward glycerol (1 m), SDS (0.1% and 1%), Cu^2+^ (1 mm and 10 mm), quinine (1 mm and 10 mm), DHCA (1 m), and acetic acid (1 and 0.1 m; Fig. 3*B*,*C*; Table 4). Mutants in *odr-3*, a Gα protein, were used as controls for these avoidance assays, since *odr-3* has been reported to be involved in multiple behaviors controlled by the ASH neurons (Roayaie et al., 1998; Hilliard et al., 2004, 2005; Zhang et al., 2016). The ASH neurons are also known to play a role in sensing mechanical stimuli (Kaplan and Horvitz, 1993). In order to test the role of SRX-97 in mechanosensation, we performed a nose touch assay with WT, *srx-97*, and *glr-1* (defective for nose touch assay; Maricq et al., 1995) animals. Upon performing the assays, we observed that *srx-97* mutant animals did not show significant defects in nose touch assays when compared with WT controls (Fig. 3*D*). These data indicate that loss of *srx-97* does not affect many aspects of the general ASH neuronal responses.

**Table 4:** Response of *srx-97* mutants toward multiple water-soluble chemicals

Water-soluble chemicals	Genotype	Avoidance in seconds
Glycerol (1 m)	WT *srx-97* *odr-3*	1.99 ± 0.43 (*n* = 30) 2.42 ± 0.17 (*n* = 33) 3.53 ± 0.25 (*n* = 30)***
SDS (1%)	WT *srx-97* *odr-3*	1.72 ± 0.23 (*n* = 30) 1.65 ± 0.18 (*n* = 36) 2.86 ± 0.21 (*n* = 32)***
Cu^2+^ (10 mM)	WT *srx-97* *odr-3*	1.13 ± 0.03 (*n* = 30) 1.10 ± 0.06 (*n* = 30) 1.53 ± 0.25 (*n* = 30)***
Dihydrocaffeic acid (100 mm)	WT *srx-97* *odr-3*	1.85 ± 0.11 (*n* = 30) 2.52 ± 0.14 (*n* = 30)** 2.36 ± 0.28 (31)**
Dihydrocaffeic acid (1 m)	WT *srx-97* *odr-3*	1.57 ± 0.21 (*n* = 32) 1.68 ± 0.32 (*n* = 31) 1.90 ± 0.15 (*n* = 31)
Acetic acid (0.1 m)	WT *srx-97* *odr-3*	3.88 ± 0.15 (*n* = 31) 3.56 ± 0.10 (*n* = 30) 5.77 ± 0.20 (*n* = 30)***
Acetic acid (1 m)	WT *srx-97* *odr-3*	2.53 ± 0.15 (*n* = 30) 2.87 ± 0.10 (*n* = 30) 4.58 ± 0.10 (*n* = 30)***
Quinine (10 mm)	WT *srx-97* *odr-3*	2.49 ± 0.20 (*n* = 35) 2.09 ± 0.21 (*n* = 30) 2.89 ± 0.16 (*n* = 32)
Quinine (1 mm)	WT *srx-97* *odr-3*	10% (*n* = 30) 9% (*n* = 30) 7% (*n* = 30)
M13 buffer	WT *srx-97* *odr-3*	6% (*n* = 30) 4% (*n* = 30) 4% (*n* = 30)

10.1523/ENEURO.0011-20.2020.f3-1 Extended Data Figure 3-1 Overexpressing SRX-97 does not affect behavior of the animals towards benzaldehyde. ***A***, Schematic of a plate showing four quadrants. The two opposite quadrants show the test spots (termed T) and the control spots (termed C), 50–150 animals are added in the central spot and the C.I. for volatile chemicals calculate by using the indicated formula. ***B***, Chemotaxis indices of the WT, *srx-97* mutant animals and overexpression lines expressing the *srx-97* gene under its endogenous promoter or the *osm-10* promoter in WT background. ***C***, Chemotaxis indices of the WT, *srx-97* mutant animals, and the rescue lines expressing *srx-97* gene tagged with mCherry under its endogenous promoter in *srx-97* mutant background. The rescue line shows nonsignificant defects when compared with WT controls or *srx-97* mutant animals. The assays in ***B***, ***C*** were done in triplicates over multiple days. Each dot in the graphs ***B***, ***C*** indicates an assay done in triplicate. The error bars represent SEM, and statistical significance is represented as “ns” for not significant; *p <* *0.05. The numbers at the base of each plot in ***B***, ***C*** indicate the number of times the experiment was performed with 50–150 *C. elegans* used in each trial. Download Figure 3-1, EPS file.

The ASH neurons are also known to be involved in detecting volatile chemicals (Troemel et al., 1995). To analyze the role of SRX-97 in detecting volatile chemicals, we used a modified chemotaxis plate, having four quadrants, two opposite quadrants for test solutions (T), and two for control solutions (C; illustrated in Extended Data Fig. 3-1*A*). Both control and test spots were 3 cm away from the *C. elegans* loading center. Before the addition of control or test solution, we added sodium azide to paralyze the animals once they reach their respective spots. Next, we calculated the C.I. by measuring the number of animals in each quadrant with the formula shown in Extended Data Figure 3-1*A*. Previous work has shown that in chemotaxis assays, the ASH neurons are involved in aversive behaviors toward the repellent 1-octanol (Chao et al., 2004). Here again, we found no significant change in the C.I. of the *srx-97* mutant line when compared with WT control animals (Fig. 3*E*). Recent findings suggest that the ASH neurons are involved in sensing high concentrations of chemicals, such as IAA (Yoshida et al., 2012) and DA (Taniguchi et al., 2014). In the chemotaxis assays, we used a range of concentrations of IAA and DA, testing for any defects in responses toward these chemicals. We found that the *srx-97* mutants did not show any significant defects in chemotaxis toward IAA and DA when compared with control animals (Fig. 3*F*; Table 5).

**Table 5:** Response of *srx-97* mutants toward volatile chemicals

Volatile chemicals	Genotype	CI index
Diacetyl (10^−2^)	WT *srx-97* *odr-3*	0.81 (*n* = 9) 0.73 (*n* = 9) 0.50 (*n* = 9)**
Diacetyl (10^−3^)	WT *srx-97* *odr-3*	0.85 (*n* = 9) 0.84 (*n* = 6) 0.70 (*n* = 6)
Isoamyl alcohol (10^−2^)	WT *srx-97* *odr-3*	0.84 (*n* = 9) 0.83 (*n* = 9) 0.78 (*n* = 9)
Isoamyl alcohol (10^−3^)	WT *srx-97* *odr-3*	0.94 (*n* = 6) 0.92 (*n* = 6) 0.87 (*n* = 6)

The ASH neurons are also known to be involved in detecting benzaldehyde (Troemel et al., 1995; Walker et al., 2009; Aoki et al., 2011; Taniguchi et al., 2014). A previously identified GPCR, DCAR-1, has homology with the SRX family of proteins and *dcar-1* mutants show defective chemotaxis toward undiluted benzaldehyde (Aoki et al., 2011). In our chemotaxis assays we found that the *srx-97* mutant animals showed significantly more attraction to a high concentration of benzaldehyde (10^−1^) when compared with WT controls animals (Fig. 3*G*). We also found that as reported previously *odr-3* mutant animals showed reduced attraction toward benzaldehyde (Fig. 3*G*; Roayaie et al., 1998). At low concentrations (10^−2^ and 10^−3^) of benzaldehyde and undiluted benzaldehyde, there was no significant difference between *srx-97* and WT animals (Fig. 3*G*). Earlier reports indicate that the ASH neurons are involved in responding to high concentrations of benzaldehyde (0.1% v/v), whereas medium or low concentrations (0.005–0.0001%) of benzaldehyde are sensed by the AWC and AWA neurons (Leinwand et al., 2015). Since SRX-97 is expressed in the ASH neurons, it could be involved in sensing a very high concentration range of benzaldehyde. In order to confirm the *srx-97* mutant phenotype, we tried to rescue the defects seen in the *srx-97* mutants. We found that the defects in chemotaxis toward benzaldehyde seen in the *srx-97* animals could be rescued by expressing SRX-97 under its endogenous promoter, and partially rescued by expressing SRX-97 under the *osm-10* promoter that drives expression in the ASH and ASI neurons (Fig. 3*H*). Although we observed rescue of the *srx-97* mutants with the *srx-97* and the *osm-10* promoter lines, we also found a small but significant rescue in animals that did not carry any observable rescuing arrays, possibly because of low expression of the rescuing array that was undetectable with the fluorescence markers in these non-array (NA) lines. Further, in the WT background, the rescuing lines of SRX-97 behaved in a manner similar to WT control animals (Extended Data Fig. 3-1*B*). We also tested the previously described mCherry-tagged SRX-97 line (Fig. 1*H*) in our rescue experiments and observed a partial rescue of the *srx-97* mutant phenotype (Extended Data Fig. 3-1*C*, the rescue line showed no significant difference with respect to WT control animals or *srx-97* mutants). This partial rescue could be by the mCherry tag hindering the function of SRX-97 or because of incomplete penetrance of expression from the arrays used. Thus far, our data suggest that the csGPCR SRX-97 is responsible for sensing high concentrations of benzaldehyde.

### Ablation of ASH causes defects in benzaldehyde sensing

**Figure 4.**
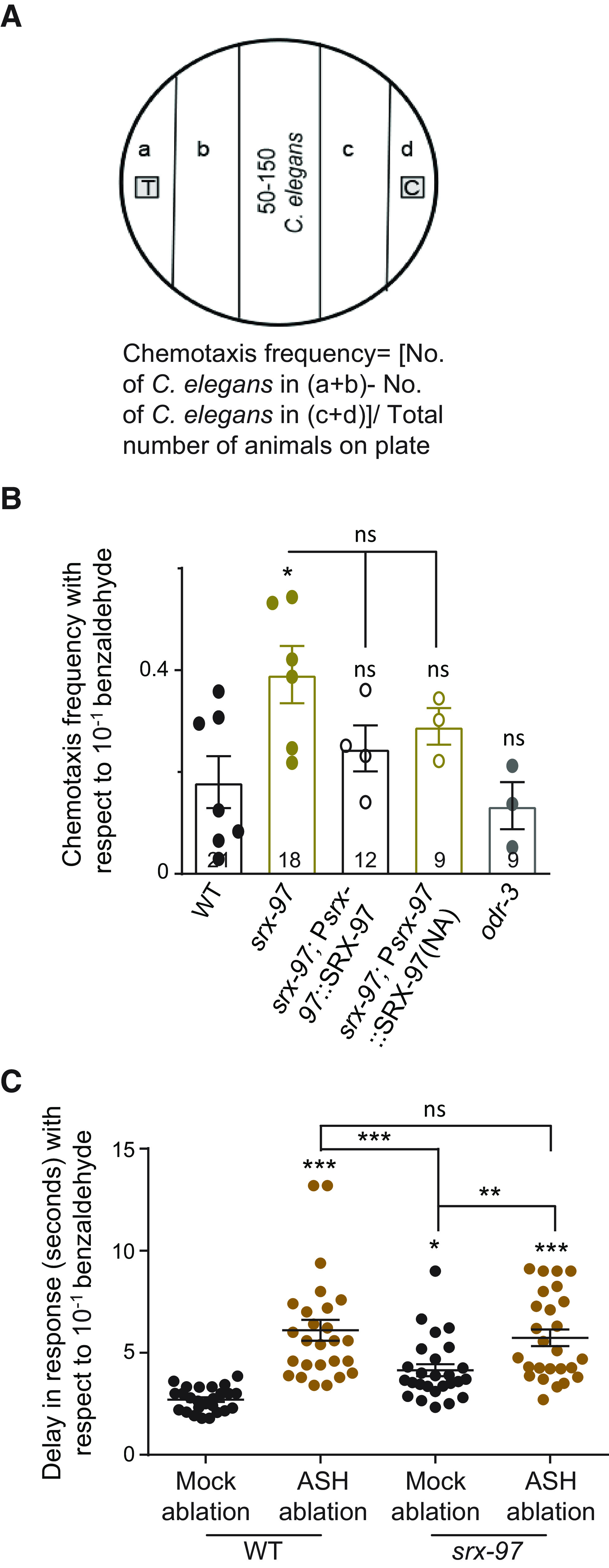
Ablation of ASH neurons shows defects toward chemosensation to benzaldehyde. ***A***, Illustration of the design of the plates used for analyzing the chemotaxis frequency of *C. elegans* along with the formula used for this calculation. Each sector (a–d) is 1 cm in width. ***B***, Graph of chemotaxis frequencies of WT, *srx-97*, the *srx-97* rescue line and a control *odr-3* mutant line to a high concentration of benzaldehyde. The assay was performed in triplicate over multiple days with each dot indicating an assay done in triplicate. The numbers at the base of each plot indicate the number of times the experiment was performed with each genotype. ***C***, Graph plotting the delay in response of animals toward a high benzaldehyde concentration. The animals used in this experiment have undergone mock ablation or ASH ablation in WT or *srx-97* mutant backgrounds. Each dot indicates a response from a single animal. Approximately 25 mock ablated animals and ASH ablated animals in WT and *srx-97* mutant background were analyzed for this experiment over multiple days. The error bars represent SEM, and statistical significance is represented as “ns” for not significant; *p <* *0.05, **p <* *0.01, ***p <* *0.001.

We next analyzed the chemotaxis frequency of *srx-97* mutants toward high concentrations of benzaldehyde (Nuttley et al., 2001). Here, we added the benzaldehyde (10^−1^) on a small sheet (0.5–1 cm in diameter) of Parafilm so it would not be soaked in the media. We also excluded the addition of sodium azide on the control and test spots so as to allow the animals to move freely toward the control or test spots. After a 60-min incubation period, the animals were counted along each sector, and the chemotaxis frequency was calculated by the formula indicated in Figure 4*A*. Again, the *srx-97* mutants showed a significant increase in their attraction toward benzaldehyde (Fig. 4*B*). This defect was reduced by expressing SRX-97 under its endogenous promoter, suggesting that SRX-97 is responsible for sensing high concentrations of benzaldehyde and that the *srx-97* phenotype may not be because of the initial attraction followed by repulsion behavior shown by the *odr-3* mutants (Fig. 4*B*; Nuttley et al., 2001). However, a similar reduction of the defect was also observed in non-transgenic siblings that could be because of low expression of the rescuing array undetectable by fluorescence (Fig. 4*B*).

In order to further strengthen our data that SRX-97 was indeed acting in ASH to sense high concentrations of benzaldehyde, we ablated the ASH neurons in WT as well as in *srx-97* mutant animals. We then tested the delay in response toward benzaldehyde in mock ablated and ASH ablated animals. Our data show that WT animals with ablated ASH neurons show a significant delay in their response to benzaldehyde when compared with the mock-ablated animals (Fig. 4*C*). Moreover, there was a significant difference in ASH ablated WT animals and mock ablated *srx-97* mutant *C. elegans*, indicating that ASH may also have other receptors that allows detection of high concentrations of benzaldehyde. Animals where the ASH neurons were ablated in *srx-97* mutants, behaved like WT animals that had undergone ASH neuron ablation, further indicating the function of SRX-97 in ASH neurons.

### Defects in sensory signaling appear to function downstream of *srx-97*

**Figure 5.**
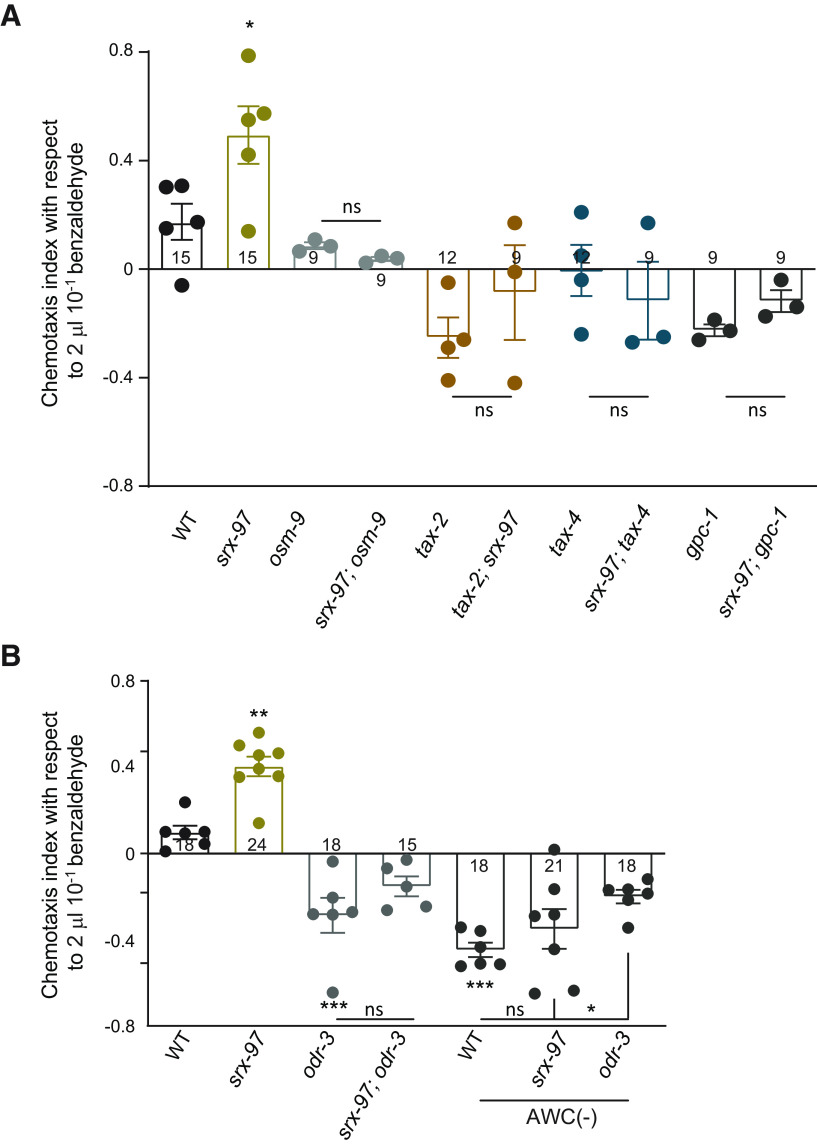
The *srx-97* mutant phenotype is suppressed by other signaling mutants that appear to function downstream of SRX-97. ***A***, Chemotaxis indices with respect to high concentration of benzaldehyde in WT, *srx-97*, *osm-9*, *tax-2*, *tax-4*, and *gpc-1* mutants along with analysis of each mutant in the *srx-97* background. ***B***, Chemotaxis indices with respect to high concentration of benzaldehyde in WT, *srx-97*, *odr-3*, and *srx-97; odr-3* mutants. This graph also indicates chemotaxis indices with respect to benzaldehyde on ablation of the AWC neuron [AWC(–)] in WT, *srx-97*, and *odr-3* mutants. The assays were performed in triplicate over multiple days. Each dot in both plots ***A***, ***B*** indicates an assay done in triplicate. The numbers at the base of each graph in ***A***, ***B*** indicate the total number of times the experiment was performed with each genotype. The error bars represent SEM, and statistical significance is represented as “ns” for not significant; *p <* *0.05, **p <* *0.01, ***p <* *0.001.

The ASH neurons express multiple GPCR associated sensory molecules that are reported to be required for signal transduction (Roayaie et al., 1998; Hilliard et al., 2004, 2005). Among these, the G-protein subunit, GPC-1 that encodes the γ subunit of GPCRs, shows a positive adaptive olfactory response toward benzaldehyde (Jansen et al., 2002; Yamada et al., 2009). We found that *gpc-1* and *srx-97* double mutants show a negative C.I. similar to what was seen with *gpc-1* mutant animals (Fig. 5*A*). These data indicated that SRX-97 could be functioning through the G-protein signaling pathway. We next tested mutants in ODR-3, a Gα protein which is primarily required for sensory signal transduction and is involved in responses toward osmotic strength, high salt concentration, nose touch, and volatile chemicals (Roayaie et al., 1998; Hilliard et al., 2004, 2005; Zhang et al., 2016). Mutants in *odr-3* have been reported to show defects in attraction toward low concentrations of benzaldehyde (1:200; Roayaie et al., 1998). In our assay, we found that *odr-3* and *srx-97; odr-3* double mutants showed negative chemotaxis indices toward high concentration of benzaldehyde, similar to what was seen with *gpc-1* mutants (Fig. 5*B*). These data further suggest that SRX-97 could be involved in chemotactic function through the GPCR pathway.

The AWC neurons sense low concentration of benzaldehyde through *odr-3* signaling (Bargmann et al., 1993). To gain more insight into the function of AWC neurons in sensing benzaldehyde, we used a line where AWC is ablated [AWC(–); Beverly et al., 2011]. We found that the loss of AWC neurons made the *C. elegans* aversive toward benzaldehyde (Fig. 5*B*). Further loss of *srx-97* in the AWC(–) background did not appear to affect the AWC(–) phenotype (Fig. 5*B*). These data suggest that AWC is the primary sensory neuron that shows attraction to diffused (in our assay) or low concentrations of benzaldehyde. Further, the ASH neurons could act as secondary sets of neurons that are responsive to high benzaldehyde concentrations as shown previously for IAA (Yoshida et al., 2012). We also found that the loss of *odr-3* in the AWC(–) animals appeared to significantly suppress the AWC ablation defects with respect to chemotaxis toward benzaldehyde (Fig. 5*B*). Studies have shown that ODR-3 is expressed in ASH, AWA, AWB and AWC neurons (Roayaie et al., 1998). Hence it is possible that ODR-3 might be affecting chemotaxis toward benzaldehyde through neurons other than AWC.

We next analyzed the molecules that may be functioning downstream of the SRX-97 GPCR. The ASH neurons express multiple channel proteins that get activated through GPCRs and are involved in regulating different behavioral outputs (Sengupta, 2007). One such molecule, OSM-9, is a member of the vanilloid subfamily of TRP channel proteins that regulates avoidance behaviors to osmotic strength, nose touch and undiluted benzaldehyde in the ASH neurons (Colbert et al., 1997; Murayama and Maruyama, 2013; Zou et al., 2017). We found that *osm-9* mutants showed a phenotype similar to that seen in WT animals when tested for a high concentration of benzaldehyde (Fig. 5*A*). The cyclic nucleotide-gated channel proteins, TAX-2 and TAX-4 are responsible for the detection of volatile chemicals like benzaldehyde by AWC and other amphid neurons, although the source of activating cGMP is still unknown (Coburn and Bargmann, 1996; Komatsu et al., 1996; Zagotta and Siegelbaum, 1996). These mutants were tested for defects in chemotaxis to benzaldehyde and showed a negative C.I. toward a high concentration of benzaldehyde (Fig. 5*A*). All three mutants, *osm-9*, *tax-2*, and *tax-4*, completely suppressed the increased chemotaxis behavior seen in *srx-97* mutant animals. The suppression of the *srx-97* mutant phenotype by these downstream molecules indicates that either the SRX-97 GPCR acts redundantly to sense the high concentrations of benzaldehyde by activating pathways different from the ones tested, or OSM-9, TAX-2, and TAX-4 function to detect both high and low concentrations of benzaldehyde and SRX-97 functions through the canonical G-protein pathway to elicit responses to high concentrations of benzaldehyde.

## Discussion

In this study, we have characterized the expression and function of the GPCR, SRX-97. From our expression studies, it is clear that SRX-97 shows expression in the ASH and PHB neurons. Moreover, the chemotaxis experiments reveal that the GPCR SRX-97 senses high concentrations of benzaldehyde. Our data indicate that in comparison with WT animals, *srx-97* null mutant animals show increased attraction toward high concentrations of benzaldehyde (10^−1^). We also show that SRX-97::mCherry driven by its native promoter shows localization along the ciliary tip of the ASH neurons. Since the cilia are the compartment where signal sensation and transduction occur, the localization of SRX-97 at the cilium tips suggests its role in sensory perception or transduction of sensory signal/s. These results suggest that SRX-97 expressed in the ASH neurons is responsible for detecting benzaldehyde from its surroundings.

Other than SRX-97, ASH neurons also express different sets of GPCRs, which sense benzaldehyde (Aoki et al., 2011; Taniguchi et al., 2014; Vidal et al., 2018). For example, DCAR-1 is expressed in ASH neurons and is involved in sensing undiluted benzaldehyde (Aoki et al., 2011). Multiple reports propose that there is a “tuning curve” for the olfactory neurons through which some olfactory receptors exhibit noticeable sensitivity (threshold) for some odorants; some neurons are activated by receptors only at low odorant concentrations, while other neurons and receptors are activated at high concentrations of odorants (Firestein, 2001; Spehr and Munger, 2009). Since the *srx-97* mutant animals show reduced but not completely abolished response toward high concentrations (10^−1^) of benzaldehyde (Figs. 3*G*, 4*C*), it is possible that SRX-97 acts as a constituent of a receptor complex on the ASH neurons allowing for detection of benzaldehyde at high or undiluted concentrations but not at low concentrations. On the contrary, low concentrations of benzaldehyde are is sensed by the AWC neurons (Bargmann et al., 1993; Leinwand et al., 2015). The WT like chemotaxis response of *srx-97* mutants toward undiluted and low concentration of benzaldehyde may suggest that animals sense their surrounding by activating different receptors using the corresponding neurons in a concentration-dependent manner and this, in turn, leads to appropriate behavioral responses.

GPCRs signal through heteromeric G-proteins signaling cascades and transduce signals from the environment through intracellular mediators that play a vital role in triggering behavior. The ASH and other amphid neurons express the Gα protein ODR-3 as well as OSM-9, a TRPV protein that is involved in the sensation of various stimuli including olfaction (Bargmann et al., 1993; Troemel et al., 1997; Roayaie et al., 1998; Hilliard et al., 2004). The amphid AWC neurons act as primary olfactory neurons involved in sensing low concentrations of benzaldehyde (Leinwand et al., 2015), while the ASH neurons are required for sensing undiluted benzaldehyde (Troemel et al., 1995; Colbert et al., 1997; Tobin et al., 2002). In our chemotaxis assay (90 min), we found that *srx-97* mutants presented chemotaxis defects toward high concentrations of benzaldehyde; in these assays the animals were placed 3 cm away from the source of benzaldehyde and hence not at short range from the source (Troemel et al., 1995). Previous work has shown that the distance or diffusion gradient of a test chemical may activate primary sensory neurons like AWA and AWC (Yoshida et al., 2012; Taniguchi et al., 2014; Leinwand et al., 2015). Our work also indicates that defects in the downstream signaling molecules in these neurons could affect the repulsion of the animals from the source.

The defects seen in the *srx-97* mutation toward DHCA is similar to the previously reported GPCR/*dcar-1* mutants expressed in ASH neurons (Aoki et al., 2011). DCAR-1 functions through *odr-3* and *osm-9* signaling pathways to elicit its response toward DHCA (Aoki et al., 2011). However, the downstream molecules for the signaling pathway remain unidentified. We also see that *srx-97* mutant animals show defects toward high osmolarity as seen in experiments using 2 m glycerol. Although in our battery of tests we saw small but significant defects in *srx-97* mutants toward 2 m glycerol and 100 mm DHCA, we do not know the mechanisms underlying these defects in the mutant animals. It will be interesting in future to understand how single GPCRs like SRX-97 and their associated proteins or downstream molecules are responsible for these different aspects of chemosensation in *C. elegans.*

Our results propose that there could be alternative pathways for signal transduction in ASH neurons through GPCRs like SRX-97. To our knowledge, downstream signaling molecules, the loss of which causes attraction to an undiluted or high concentration of benzaldehyde through the ASH neurons have not yet been identified. The *C. elegans* genome encodes for 21 Gα, two Gβ, and two Gγ genes (Jansen et al., 1999; Cuppen et al., 2003). Out of these, 11 Gα proteins are expressed in the ASH neurons (Bastiani and Mendel, 2006). Our results suggest that the ASH neurons are involved in aversion to undiluted or high concentrations of benzaldehyde through multiple or redundant chemosensory pathways involved in the signaling through GPCRs like SRX-97.

In conclusion, our results bring out the possibility that SRX-97 is a key mediator in chemotaxis toward high concentrations of benzaldehyde in the chemosensory system of *C. elegans*. However, the downstream signaling components still need to be identified, to provide more details into the functioning of SRX-97 in the ASH neurons. These investigations may offer insights into the nature of signal transduction in ASH neurons and their physiological role in concentration-dependent avoidance responses.

## Synthesis

Reviewing Editor: Douglas Bayliss, University of Virginia School of Medicine Decisions are customarily a result of the Reviewing Editor and the peer reviewers coming together and discussing their recommendations until a consensus is reached. When revisions are invited, a fact-based synthesis statement explaining their decision and outlining what is needed to prepare a revision will be listed below. The following reviewer(s) agreed to reveal their identity: Sreekanth Chalasani.

This revised manuscript was seen by the original two reviewers, who appreciate that this work identifies a new G protein-coupled receptor for a volatile compound in C. elegans, and felt that the responses and revisions had (mostly) addressed the previous concerns. Nevertheless, some additional points have come to light with the new information that was presented with the revision. Some of these points relate simply to the presentation (e.g., image quality, jargon), whereas others suggest inclusion of data, caveats, and clarifications that affect interpretations and conclusions (e.g., tagged SRX-97::mCherry is non-functional; receptor selectivity issues; inconsistent phenotype relative to the initial aldicrab screen; etc.). These and the other remaining concerns (provided below) should be addressed in a revised manuscript.

Please find the full comments appended below.

----

Reviewer #1

Advances the Field

It does advance the field as it identifies a new receptor for a volatile compound in the nematode C. elegans.

Comments

The manuscript eN-NWR-0011-20R1 entitled “The G-protein coupled receptor SRX-97 is required for concentration dependent sensing of Benzaldehyde in Caenorhabditis elegans", has been considerably revised and provides now more compelling arguments for the conclusion that the newly identified G-protein coupled receptor SRX-97, is expressed in ASH and PHB sensory neurons, localizes to the ASH sensory cilia, and is a putative receptor of high concentration of benzaldehyde, which mediates repulsion to this chemical. The authors are to be commended for having made a number of important revisions. There are however still a few issues that needs to be addressed.

1. In Figure 1 Panel F, the authors present the localization of the SRX-97::mCherry. The images are cryptic and currently impossible to study. Even without using confocal microscopy, the authors should provide images of sufficient clarity and contrast, and should flank them by a scheme to reveal what section of the animal the reader is looking at. This is especially important for non-expert in this model organism.

2. The authors disclose in the rebuttal that the tagged SRX-97::mCherry is not a functional molecule, as it cannot rescue the srx-97 mutant phenotype. How can they be sure then that the observed localization is correct? Have they tried multiple transgenic lines to see if there are some that can rescue the srx-97 mutant phenotype? At a minimum, this non-functional information on SRX-97::mCherry needs to be clearly stated in the paper, when the transgenic strain is presented.

3. In this revised version, following the reviewers’ request of clarification, the authors mention that SRX-97 was originally identified from an RNAi screen for aldicarb resistance/hypersensitivity. However, when they tested the CRISPR SRX-97 they could not reproduce the aldicarb effect, and conclude/state that the RNAi results might have been a false positive. Have they reexamined and confirmed whether RNAi of SRX-97 does not indeed cause any aldicarb effect? At this regard, they use a 2 kb promoter to determine the expression pattern of SRX-97 and define that it is restricted to the ASH and PHB neurons. Have they tested a longer promoter of 3 or 5 kb, or included the first intron of the gene, that could reveal expression in other tissues? Did they test if the strain with overexpression under the endogenous promoter, which they have generated, causes any aldicarb phenotypes? Some of these experiments might conclusively clarify the specificity of expression and function of SRX-97.

4. The overexpression results related to the effect on ASH, or lack thereof, should be included in the manuscript.

5. On page 20, lines 314 - 316 the authors state that srx-97 mutant present some defects in avoidance of 2 M Glycerol (now presented in Fig 3A) and 100 mM dihydrocaffeic acid (Table 4). How does this reconcile with the specificity of this receptor for high concentration of benzaldehyde? This should be discussed in the paper as it is possible that the receptor is activated also by these stimuli.

6. The paper would benefit to have the data on nose touch response in Fig 3, in the top panels, as it strengthens the idea that srx-97 does not affect to a great extent the general ASH response.

7. On page 20 in lines 319-321, and in Table 4, the authors state that the response of SRX-97 mutants to Quinine 1 mM is not different from wt. However, the wt response they report is not different from the buffer. Does it mean that this assay did not work in the first place as Quinine did not function as a repellent for the wt?

8. Line 58-59 on page 3. The authors state that “The ASH neurons convey information through multiple receptors. For example, touch has been shown to be detected by mechanically gated ion channels MEC-4 and MEC-10, while hyperosmolarity is detected by OSM-10 (Hart et al., 1999).” It is incorrect that mec-4 and mec-10 are responsible for detection in ASH, rather in the mec neurons, and this should be revised.

9. In the manuscript they use singular to refer to ASH neurons. This should be revised.

10. In Figure 4 Panel D, the statistical comparisons between groups are not clear and can be better represented with horizontal lines. Most importantly, did the authors compare ASH ablation in WT vs mock ablation in srx-97? These data sets seem different. If srx-97 is mediating the benzaldehyde response within ASH, does this mean that there is another receptor in ASH that can also affect the response to this chemical? Please discuss.

11. In Figure 3, did the authors compare the Posm-10::SRX-97 with Posm-10::SRX-97(NA)? Are they significantly different? Please explain.

12. Colloquial language is still used. Terms such as small defects (line 314) and picked it up (line 306) should be avoided.

13. The gene names and C. elegans should be italics in the references (point 11 minor comments).

14. Several typos and inconsistencies are still present in the text and have been provided on a revised PDF for simplicity.

Reviewer #2

Advances the Field

The paper is much improved now - I think they adequately responded to all concerns and have now met the standard for publication.

Comments

## Author Response

We thank the Reviewing Editor, Douglas Bayliss and the Reviewers’ for their comments that have improved our manuscript and we have tried our best to respond to the points raised by the Reviewer.

The Reviewer has brought up some good points and we appreciate the opportunity to improve the presentation of our data to make it more convincing.

In response to the Reviewer comments regarding control experiments using just the overexpression line, performing imaging with a second promoter construct to confirm the localization of srx-97 and rescue of the srx-97 phenotype using the tagged line, we have performed these experiments and added the same to the manuscript. This data has been added to Figure 1 and Figure 3-1. We have also edited the

manuscript to improve the style and grammar of the manuscript and correct the typographical errors.

These new results, as well as the complete response to the Reviewer comments are detailed below. We have gone through the comments very carefully, and are now submitting a revised manuscript that we believe takes into consideration the concerns of the Reviewer. The changes made to the manuscript are in blue in a file not containing the title page. The line numbers indicated in this document are based on the changed manuscript with no title page.

We hope that the Editor and Reviewer agree that these changes have addressed their concerns and that our manuscript is now acceptable for publication in eNeuro.

Response to Reviewer #1

Comments

The manuscript eN-NWR-0011-20R1 entitled “The G-protein coupled receptor SRX-

97 is required for concentration dependent sensing of Benzaldehyde in Caenorhabditis elegans", has been considerably revised and provides now more compelling arguments for the conclusion that the newly identified G-protein coupled receptor SRX-97, is expressed in ASH and PHB sensory neurons, localizes to the ASH sensory cilia, and is a putative receptor of high concentration of benzaldehyde, which mediates repulsion to this chemical. The authors are to be commended for having made a number of important revisions. There are however still a few issues that needs to be addressed.

We thank the Reviewer for their comments and are happy that they felt that we had largely addressed their concerns. We have tried to address the remaining issues as detailed below along with corresponding changes to our manuscript.

1. In Figure 1 Panel F, the authors present the localization of the SRX-97::mCherry. The images are cryptic and currently impossible to study. Even without using confocal microscopy, the authors should provide images of sufficient clarity and

contrast, and should flank them by a scheme to reveal what section of the animal the reader is looking at. This is especially important for non-expert in this model organism.

We have tried to have increased contrast in SRX-97::mCherry image and have outlined and flanked the section of the image represented for greater clarity as indicated in Figure 1G.

2. The authors disclose in the rebuttal that the tagged SRX-97::mCherry is not a functional molecule, as it cannot rescue the srx-97 mutant phenotype. How can they be sure then that the observed localization is correct? Have they tried multiple transgenic lines to see if there are some that can rescue the srx-97 mutant phenotype? At a minimum, this non-functional information on SRX-97::mCherry needs to be clearly stated in the paper, when the transgenic strain is presented.

Thank you for this comment. We have repeated the experiment with different lines and see partial rescue in of the srx-97 mutant phenotype. We have added this data in figure 3-1C and have discussed this data in lines 374- 380.

3. In this revised version, following the reviewers’ request of clarification, the authors mention that SRX-97 was originally identified from an RNAi screen for aldicarb resistance/hypersensitivity. However, when they tested the CRISPR SRX-97 they could not reproduce the aldicarb effect, and conclude/state that the RNAi results might have been a false positive. Have they reexamined and confirmed whether RNAi of SRX-97 does not indeed cause any aldicarb effect? At this regard, they use a 2 kb promoter to determine the expression pattern of SRX-97 and define that it is

restricted to the ASH and PHB neurons. Have they tested a longer promoter of 3 or 5 kb, or included the first intron of the gene, that could reveal expression in other tissues? Did they test if the strain with overexpression under the endogenous promoter, which they have generated, causes any aldicarb phenotypes? Some of these experiments might conclusively clarify the specificity of expression and

function of SRX-97.

Thank you for these suggestions. Due to the presence of upstream gene around 600 bp from the start of the srx-97 gene we are not able to make the 3-5 kb promoter

(this would encompass the entire upstream gene) construct. We have generated a

shorter promoter (600 bp) between srx-97 and its upstream gene and again exclusively found srx-97 expression in a pair of amphid and phasmid neurons. We have added this data to Figure 1C and lines 255- 256.

We have added the aldicarb data at the end of this document (not in the manuscript). This graph shows that the srx-97 mutants that we have generated do not show an aldicarb phenotype. We also do not see any aldicarb phenotype with the SRX-97 overexpression line. We have not redone the RNAi experiment, but agree that it will be a good idea to do so in the future. Adding aldicarb data to this manuscript could

be confusing as it would stray from the theme of this manuscript, which describes the chemotaxis defects in srx-97 mutants. We have however added the following to line “The aldicarb phenotype in the RNAi screen could have been a false positive due to cross-complementation reactions with other GPCRs” (lines 305- 306). I hope you will agree with us on this point.

4. The overexpression results related to the effect on ASH, or lack thereof, should be included in the manuscript.

Thank you for this suggestion. We have included this data in Figure 3-1B and lines

373- 374.

5. On page 20, lines 314 - 316 the authors state that srx-97 mutant present some defects in avoidance of 2 M Glycerol (now presented in Fig 3A) and 100 mM dihydrocaffeic acid (Table 4). How does this reconcile with the specificity of this receptor for high concentration of benzaldehyde? This should be discussed in the paper as it is possible that the receptor is activated also by these stimuli.

Thank you for this suggestion. Yes on the basis of our results, along with benzaldehyde srx-97 mutants appears to show a differential response towards 2 M Glycerol and 100 mM dihydrocaffeic acid. We do not know how this activation could occur; it may be due to just the SRX-97 GPCR or other associated molecules. We have discussed this point in lines 516- 521 and added this point to the conclusion (lines 533- 534).

6. The paper would benefit to have the data on nose touch response in Fig 3, in the top panels, as it strengthens the idea that srx-97 does not affect to a great extent the general ASH response.

Thank you for the suggestion. We have now added the data as a graph in figure 3D

and discussed these results in lines 329- 330.

7. On page 20 in lines 319-321, and in Table 4, the authors state that the response of SRX-97 mutants to Quinine 1 mM is not different from wt. However, the wt response they report is not different from the buffer. Does it mean that this assay did not work in the first place as Quinine did not function as a repellent for the wt?

Thank you for pointing this out. We have repeated the experiment to better understand the effect of 1 mM Quinine on WT and srx-97 mutants. We did find there is a very small effect on WT animals with respect to the buffer control and no significant effect of 1 mM quinine on srx-97 mutants when compared to WT animals (Table number 4)

8. Line 58-59 on page 3. The authors state that “The ASH neurons convey information through multiple receptors. For example, touch has been shown to be detected by mechanically gated ion channels MEC-4 and MEC-10, while hyperosmolarity is detected by OSM-10 (Hart et al., 1999).” It is incorrect that mec-4 and mec-10 are responsible for detection in ASH, rather in the mec neurons, and this should be revised.

Thank you very much for pointing out this mistake, we have corrected this error in our manuscript (lines 58- 60).

9. In the manuscript they use singular to refer to ASH neurons. This should be revised.

Thank you for pointing out this error. We have made the change to ASH neurons throughout the text.

10. In Figure 4 Panel D, the statistical comparisons between groups are not clear

and can be better represented with horizontal lines. Most importantly, did the authors compare ASH ablation in WT vs mock ablation in srx-97? These data sets seem different. If srx-97 is mediating the benzaldehyde response within ASH, does this mean that there is another receptor in ASH that can also affect the response to this chemical? Please discuss.

Thank you very much for pointing this out. We do find a significant difference in ASH ablation in WT vs mock ablation in srx-97 mutant animals. Our results indicate the SRX-97 is not the sole mediator of benzaldehyde response in ASH neurons and there are likely other receptors for benzaldehyde in the ASH neuron. These results are described in lines 401- 404 and discussed in lines 482- 487.

11. In Figure 3, did the authors compare the Posm-10::SRX-97 with Posm-10::SRX-

97(NA)? Are they significantly different? Please explain.

Thank you. We have found this the comparison between Posm-10::SRX-97 and Posm-10::SRX-97(NA) to be statistically non-significant indicating only a partial rescue of this phenotype. We have explained this in lines 366- 373.

12. Colloquial language is still used. Terms such as small defects (line 314) and picked it up (line 306) should be avoided.

Thank you. We have modified the colloquial language throughout the manuscript.

13. The gene names and C. elegans should be italics in the references (point 11 minor comments).

Thank you and we apologize for having left this out in the last revision. We have changed C. elegans and the gene names to italics in the references.

14. Several typos and inconsistencies are still present in the text and have been provided on a revised PDF for simplicity.

Thank you. We have gone through the manuscript and modified the typos and inconsistencies throughout the text.

Response to Reviewer #2

Advances the Field

The paper is much improved now - I think they adequately responded to all concerns and have now met the standard for publication.

We thank the Reviewer.
